# High quality Chromosome-scale Genome Assembly of *Phlebotomus perniciosus*, a Vector of Zoonotic Leishmaniasis

**DOI:** 10.1038/s41597-026-07617-1

**Published:** 2026-07-01

**Authors:** Joseph Thorpe, Nina Billows, Tomas Becvar, Emma Collins, Matthew Higgins, Myrela Santos de Jesus, Mary Cameron, Carla Maia, Petr Volf, Taane G. Clark, Susana Campino

**Affiliations:** 1 Faculty of Infectious and Tropical Diseases, London School of Hygiene and Tropical Medicine, London, UK; 2 Department of Parasitology, Faculty of Science, Charles University, Prague, Czech Republic; 3 Global Health and Tropical Medicine, GHTM, LA-REAL, Instituto de Higiene e Medicina Tropical, IHMT, Universidade NOVA de Lisboa, Lisboa, Portugal; 4 Faculty of Epidemiology and Population Health, London School of Hygiene and Tropical Medicine, London, UK

**Keywords:** Genetics, Genetic variation, Genome informatics

## Abstract

*Phlebotomus perniciosus* is a major sand fly in the Western Mediterranean and a primary vector for *Leishmania infantum*, causing human and canine visceral leishmaniasis. This species can also transmit phleboviruses, including Toscana virus, associated with meningitis and encephalitis. Despite its importance, genomic resources for *P. perniciosus* remain limited, with only a low-quality assembly available. A high-quality reference genome is essential to study traits related to vector competence, host preference, and responses to control measures. Here, we present a high-quality de *novo* assembly using Oxford Nanopore long-read sequencing. We evaluated two assembly strategies, guided and unguided, using Canu and Flye. The guided Canu assembly was optimal, producing a 143 Mb genome with scaffold N50 of 48 Mb and 95% BUSCO completeness. Annotation with RNA-seq and protein homology predicted 12,492 genes (96% BUSCO) and 24% repetitive sequences. The complete mitochondrial genome was also assembled. This robust genome offers a foundation for investigating vector biology and adaptive traits, expanding genomic resources for phlebotomine sand flies and supporting improved leishmaniasis surveillance and control efforts.

## Background & Summary

*Phlebotomus perniciosus* is a hematophagous (blood-feeding) sand fly species (Diptera: Psychodidae: Phlebotominae). This species is responsible for the transmission of the protozoan *Leishmania infantum* and several phleboviruses and may also serve as ecological indicator due to its sensitivity to environmental changes^1,2^. Traditionally, *P. perniciosus* has been confined to regions of the western Mediterranean, where it thrives in mild, temperate climates^3^. However, recent reports indicate an ongoing expansion of its geographical range, including into areas with cooler climates and higher altitudes. This shift is attributed to a combination of factors, including climate change^4^, urbanisation, land-use changes, and the movement of domestic animals, particularly dogs, which act as reservoir hosts for *Leishmania infantum*^5^. These ecological and anthropogenic changes raise significant public health concerns, as they may facilitate the emergence and transmission of leishmaniasis in areas previously considered non-endemic.

Understanding the ecology, behaviour, and vector competence of *P. perniciosus* is crucial for developing targeted strategies to control leishmaniasis in endemic areas. Despite its epidemiological significance, the Phlebotominae subfamily remains underrepresented in genomic databases^6,7^. The current available genomes only include, *P. papatasi* and *P. argentipes*, and an existing low-contiguity *P. perniciosus* genome. There are also other sand fly genomes available for other genera and species within the subfamily, such as for *Lutzomyia longipalpis* and *Sergentomyia squamirostris*, the latter being a more fragmented assembly^8,9^.

A high-quality reference genome for *P. perniciosus* is crucial for identifying the genetic factors underlying its vectorial capacity, including host preference, immune function, and interactions with parasites and viruses^10^. It can also inform novel vector control strategies, support population and functional genomics, and facilitate tools like CRISPR and RNAi^11,12^.

This study aims to generate a *de novo* assembly of the *P. perniciosus* genome using Oxford Nanopore Technology (ONT), based on high-molecular-weight DNA extracted from individual specimens of a colony established from females originally collected in Murcia, Spain, in order to produce the first high-quality genomic resource for this species. We assess the quality of the genome assembly and its annotation and compare it to other sand fly genomes within the Phlebtominae subfamily. Our analysis reveals strong genomic synteny with closely related species and successfully identifies the mitochondrial genome of this previously uncharacterised species. This reference genome provides a critical foundation for investigating the biology, population diversity, and evolutionary dynamics of *P. perniciosus*, as well as for informing novel strategies to control *L. infantum* transmission. In addition, genomic data can strengthen surveillance and monitoring efforts by enabling accurate species identification, tracking population movements, detecting shifts in vector competence or geographic range, and supporting the development of molecular diagnostics and resistance monitoring tools.

## Methods

### Sample collection and sequencing

Samples were obtained from a colony established from females originally collected in Murcia, Spain, and maintained at Charles University, Prague, under standard conditions since 2007^13,14,15^. Individual sand flies were homogenised using a pestle and DNA was extracted using Dynabeads™ SILANE Genomic DNA Kit using the manufacturer’s instructions, to produce high genomic weight DNA. The concentration of the DNA was assessed using dsDNA high sensitivity Qubit assay with Fluorometer. The 4200 TapeStation system, with Genomic DNA ScreenTape assay (Agilent), was used to assess the quality and quantity of DNA, and the sample with the highest molecular weight DNA was selected (80% DNA with peak at 20,000 bp).

A DNA library was prepared using the Ligation Sequencing Kit V14 (SQK-LSK114; Oxford Nanopore Technologies (ONT), UK), following the manufacturer’s protocol. The DNA library was sequenced on an R10.4.1 PromethION flow cell (FLO-PRO114M; ONT, UK). Raw reads were basecalled using Dorado v0.9.1 super high accuracy model (dna_r10.4.1_e8.2_400bps_sup@v4.3.0) to obtain raw reads in FASTQ format from POD5 files. The -–trim ON option was used to remove adaptor sequences which were subsequently checked using the NCBI Foreign Contamination Screen (FCS) screening tool. Short-read Illumina sequencing (150 bp paired-end reads) was also generated by Eurofins Genomics on samples from the same laboratory colony, as well as from a wild-type specimens collected in the Algarve region, Portugal.

### Raw read quality control

Kraken2 (v2.1.3)^16^ was used to assign taxonomic classifications to the raw ONT reads to identify potential contaminants. Bacterial reads identified by Kraken2 were removed using KrakenTools (extract_kraken_reads.py). The remaining reads were scrubbed and filtered using yacrd^17^ to exclude chimeric sequences, whilst chopper software^18^ was applied to retain reads with a minimum length of 1000 and quality score of 12, thereby improving assembly quality.

### *De novo* assembly

*Phlebotomus perniciosus* was assembled using an established pipeline (available at https://github.com/MatthewHiggins2017/DenovoAssemblyTools/). High-quality reads underwent three assembly approaches using Canu^19^ and Flye^20^ software. Assemblies were iteratively refined with different read error rates, with a final accepted error rate of 0.06 for downstream analysis. Unguided scaffolding was performed using ntLink^21^ with the run_rounds_gaps option to reduce assembly gaps. The assemblies diverged at this point: Canu and Flye assemblies proceeded with guided assembly, while the unguided assembly skipped this step and proceeded directly to gap closing. Guided assemblies used RagTag software^22^, employing reference genomes *from P. perniciosus* (GCA_918844115.2)*, P. papatasi* (GCF_024763615.1), *P. argentipes* (GCF_947086385.1), and *Lu. longipalpis* (GCF_024334085.1) to assess the most effective guide. *P. argentipes* and *P. perniciosus* references performed poorly due the number of scaffolds and low assembly quality. *Lu. longipalpis* was excluded as a guide due to its four-scaffold assembly, conflicting with the expected five chromosomes of *P. perniciosus*^23,24^. The *P. papatasi* genome produced the highest quality guided assembly. Gap filling was performed using TGS-GapCloser^25^, and all three assemblies were polished using Racon^26^.

### Genome quality assessment

Genome size was estimated using Jellyfish^27^. GenomeScope software^28^ was used for k-mer plot visualisation (Supplementary Fig. 1). BUSCO^29^ assessments were performed at each assembly step using the diptera_odb10 database to monitor improvements (Supplementary Fig. 2). BUSCO scores were compared across assemblies and with existing genomes to evaluate assembly completeness and quality.

### Post-assembly optimisation

Further optimisation improved assembly quality. Application of purge_dups software reduced the duplication rate in the initial Canu assembly from 20% to 1.3%^30^. The duplication rate in the Flye assembly was reduced from 6% to 1.1% using the same purge_dups tool. Assemblies were further refined and base-level accuracy assessed using short-read Illumina data generated from *P. perniciosus colony* samples and wild type samples collected in Portugal. Polishing with Polypolish^31^ led to reduced single nucleotide polymorphism (SNP) counts and improved read mapping statistics. The Illumina reads were aligned to the assembled genome using BWA^32^. SAMtools^33^ and SNPeff^34^ tools were used to analyse the coverage and number of SNPs in the dataset. Repeat masking was conducted using RepeatMasker^35^ and RepeatModeler^36^ tools, with soft-masked genomes used in subsequent analyses.

### Gene annotation

Genome annotation was predicted using multiple strategies to ensure high accuracy. RNA-seq data from the European nucleotide archive (ENA) were used to improve annotations. Liftoff^37^, GALBA^38^, BRAKER^39^, and EGAP pipelines were tested, with EGAP providing the highest quality annotation, integrating RNA-seq and protein homology evidence. Additional annotation steps included tRNA identification with tRNAscan-SE^40^, and rRNA detection using Barrnap^41^, both incorporated into the final genome annotation. Annotation completeness was evaluated using BUSCO methodology, employing the diptera_odb10 database in -m mode. Protein sequences were clustered with CD-HIT^42^ at 100% identity to minimise redundancy, retaining a single representative per sequence for downstream analyses.

### Gene and nucleotide synteny

Gene synteny was assessed using JVCI^43^ and MCscan^44^, comparing coding sequences of the assemblies to the *P. papatasi* reference genome. Nucleotide synteny was assessed using NUCMER^45^, and homologous regions were identified with the show-coords tool. Visualisation was performed using pycircos^46^.

### Identification of orthologs and evolutionary relationships

Orthologs were identified using OrthoFinder^47^, employing protein sequences *from P. papatasi, Lu. longipalpis, P. argentipes*, and *S. squamirostris*. Phylogenetic relationships were inferred within the OrthoFinder pipeline using MAFFT^48^ for alignment and FastTree^49^ for tree construction. The resulting tree was visualised with iTOL^50^.

### Assembly of the mitochondria

The mitochondrial genome was assembled using multiple approaches. The *P. papatasi* mitochondrial reference sequence (NC_028042.1) was extracted using seqkit^51^ and used to identify homologous reads in the ONT dataset. Raw long reads were converted from FASTQ to FASTA using seqtk^52^, and a BLAST^53^ database was constructed with makeblastdb. Homologous reads were identified with blastn, filtered for uniqueness, and extracted from the original FASTQ using seqtk subseq. The filtered reads were assembled into a single circular contig using Flye, producing a 15 kb mitochondrial genome. Annotation and visualisation were performed using MitoZ^54^.

## Data Records

This Whole Genome Shotgun project has been deposited at DDBJ/ENA/GenBank under the accession GCA_056882495.1. The version described in this paper is version GCA_056882495.1^55^.

The raw sequencing data for *P. perniciosus*, including Illumina and ONT reads, are deposited in NCBI GenBank under accession code PRJNA1400036, they are available under the accession numbers SRR37574185 for long reads, and SRR36732761-SRR36732762 for short read data^14^. The whole-genome assembly annotation files have been deposited in a Zenodo repository and are publicly available for download^56^.

## Technical Validation

### Quality evaluation of the assembly and the annotation

High-quality ONT data for the *P. perniciosus* genome were assembled using both Flye and Canu to enable comparative evaluation. Both assemblers produced similar results, including comparable genome sizes and N50 values, closely matching the estimated genome size (143 Mb; Supplementary Fig. 1) and aligning with genome sizes reported for other sand fly species, such as *Lu. longipalpis* (147 Mb).

The Flye assembly achieved 94.7% BUSCO completeness, with 3,075 out of 3,285 orthologous genes identified (Table 1, Supplementary Fig. 2). The unguided assembly produced a modest N50 of 2 Mb, whereas the guided assemblies achieved significantly higher N50 values of 47 Mb and 48 Mb, respectively. The primary difference between the assemblers was scaffold count, with Canu software generating 76 scaffolds and Flye producing 223. Both assemblies contained minimal gaps, with gap contents of 0.025% and a difference of just 0.008% between them (Supplementary Table 1).

**Table 1:** Assembly statistics of all assemblies.

Assembly statistics	*P. perniciosus* (old) *	*P. perniciosus* (flye new guided)	*P. perniciosus* (flye new unguided)	*P. perniciosus (canu new guided)*	P. papatasi **	Lu. longipalpis***
Genome size	166 Mb	142 Mb	155 Mb	142 Mb	351 Mb	147.8 Mb
Scaffold N50	14Kb	48 Mb	2 Mb	47 Mb	111 Mb	40.6 Mb
Scaffold count	77886	223	1105	76	645	4
Percentage gaps	0.226%	0.017%	0.8%	0.025%	0.1%	0.082%
GC content	37%	37.59%	37.6%	37.61%	34%	35.5%
BUSCO score (out of 3285)	87.3%	94.7%	90.8%	94.4%	94.2%	95.3%
Complete and single copy BUSCOs	2812	3075	2972	3059	3056	3107
Complete and duplicated	55	36	136	42	40	24
Missing	204	137	140	140	142	130

^*^GCA_918844115.2, **GCF_024763615.1, ***GCF_024334085.1.

The base-level assembly accuracy was high, with 95% of the Illumina sequencing reads of *P. perniciosus* samples being successfully mapped to the reference of both the Flye and Canu assemblies. The number of SNPs identified was also indicative of a good genome assembly, with Canu detecting 1,238,790 SNPs and 482,290 indels, while Flye found 1,262,176 SNPs and 494,315 indels. To provide further context, the guided reference from the ENA contained 6,593,880 SNPs (approximately 1 SNP per 53 bp), compared to 1 SNP every 112 bp for Flye and every 114 bp for Canu. The Canu and Flye assemblies were highly similar, exhibiting comparable high-quality SNP profiles and sequencing coverage.

The quality of the gene annotation was assessed using BUSCO, which identified 96.2% of conserved genes (3,177 out of 3,285) in the predicted gene set, indicating high annotation completeness. The total number of genes identified was similar across both assemblies (Canu 12,492 vs. Flye 12,291; Table 2). Minor differences in gene structure metrics, such as average gene length and exon count, likely reflect assembly-specific variation or sensitivity differences between annotation tools (Table 2).

**Table 2:** Genome annotation statistics across *Phlebotomus perniciosus* *de-novo* assemblies and *P. papatasi* reference species.

	Canu	Flye	*Phlebotomus papatasi*
BUSCO score (proteins)	96.5%	96.7%	96.3%
Complete BUSCOs	3169	3177	3162
Duplicated BUSCOs	151	152	874
Fragmented BUSCOs	12	9	10
Missing BUSCOs	104	99	113
No. Genes	12492	12291	12605
mRNAs*	17962	17887	20318
lncRNAs**	1294	1238	549
RNA	271	185	56
rRNAs***	50	28	173
tRNAs***	57	149	221
Transcripts	17	33	495

Comparison of BUSCO completeness, gene content, and RNA features between Canu and Flye assemblies of *P. perniciosus* and the *P. papatasi* reference genome.

Calculated using agat software and BUSCO software.

*messenger RNA. **Long non-coding RNA. *** ribosomal RNA. **** transfer RNA.

### Synteny analysis

**Fig. 1:**
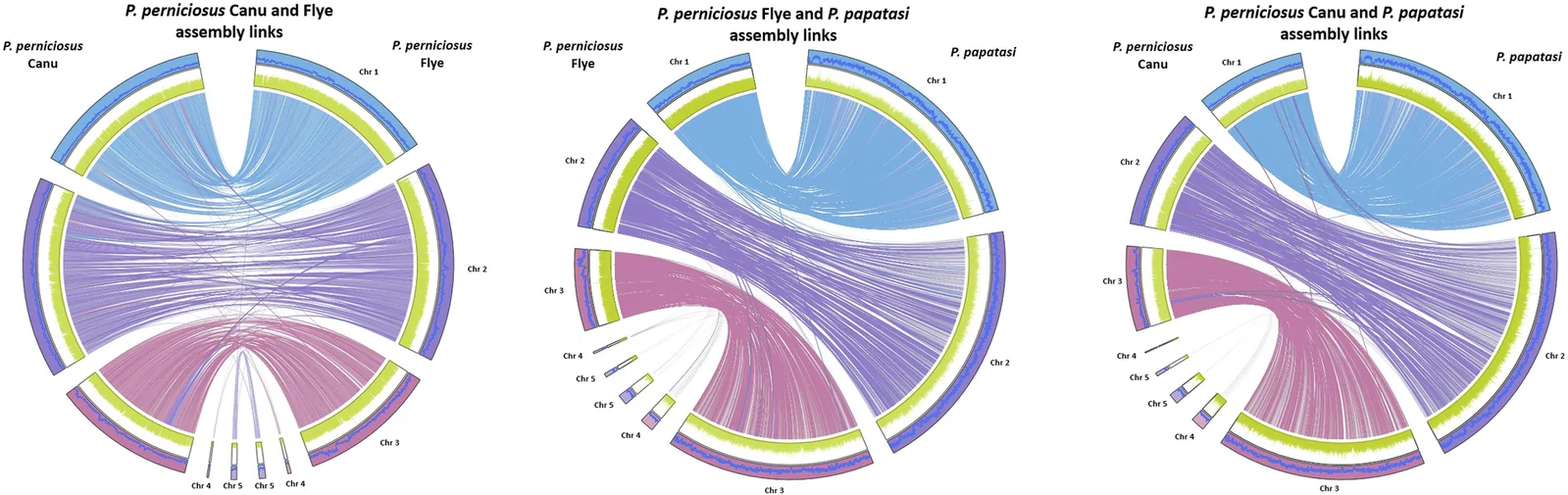
Comparative genome assemblies of *Phlebotomus perniciosus* using Flye and Canu, with guided alignment to *P. papatasi*. Nucleotide links between (**A**) *P. perniciosus* two assemblies, (**B**) The guided reference *P. papatasi* against *P. perniciosus* Flye assembly, (**C**) The guided reference *P. papatasi* against *P. perniciosus* Canu assembly. The links are coloured according to the colour of the chromosome the link is from. The coverage of the mapped files to the assembly are identified by the yellow markings underneath the chromosome. The SNP coverage across the chromosomes is identified by the squiggle within each scaffold.

Both genomes underwent nucleotide and gene synteny analysis between the *P. papatasi* guided reference and the *P. perniciosus* genome assembly to evaluate assembly structure and accuracy. Additionally, synteny analysis was performed between the Flye and Canu assemblies to assess their concordance (Fig. 1). The nucleotide-level synteny revealed a high degree of collinearity between the Flye and Canu assemblies, and importantly, between the *P. papatasi* reference and the *P. perniciosus* assembly. The strong consistency, with only minor discrepancies, highlights the high quality of the resulting assembly.

**Fig. 2:**
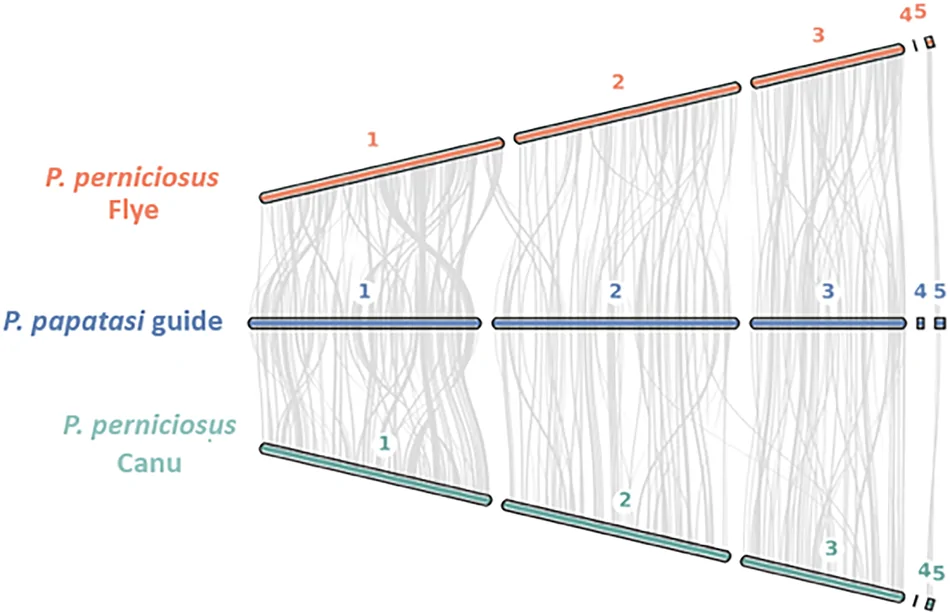
Gene synteny analysis. The Canu and Flye assemblies, compared to the guided reference of *P. papatasi*. Gene links are identified with the grey lines, across 5 comparable chromosomes.

Gene synteny was evaluated through a comparative analysis of the Flye and Canu assemblies, focusing on the conservation of gene order relative to the guided reference (Fig. 2). While a large proportion of genes (96.4%) adhered to the expected reference pattern, several notable discrepancies were observed, particularly on chromosome 1, where multiple genes were located in non-corresponding positions. Overall, the Canu assembly showed stronger concordance with the guided reference, with a higher number of genes correctly assigned to their respective chromosomes and substantially fewer mismatches than the Flye assembly.

### Comparison of orthologs

**Fig. 3:**
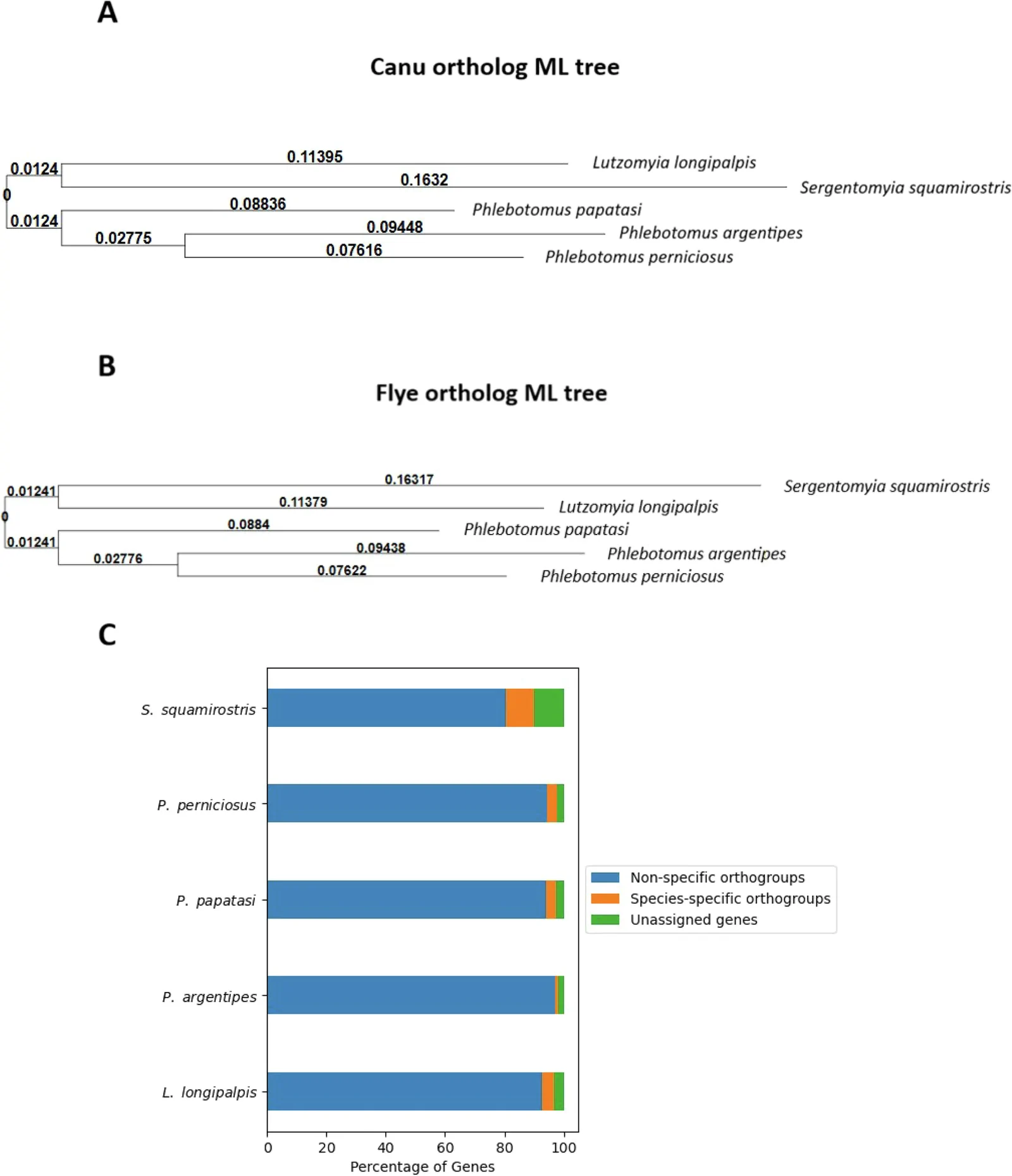
Ortholog analysis of Flye and Canu assemblies.

A detailed comparison of the annotated genomes was conducted to examine gene evolution and duplication events (Table 3). A high proportion of genes (97.7%) were assigned to orthogroups, indicating strong representation of conserved gene families across the assemblies. The total number of orthogroup-assigned genes in *P. perniciosus* (17,557) was comparable to *P. argentipes* (15,398) and *P. papatasi* (19,648), reflecting consistent annotation completeness. This high level of orthogroup assignment and conserved gene content underscores the robustness of the genome annotation and suggests strong evolutionary conservation within the *Phlebotomus* genus. To further explore evolutionary relationships, a maximum likelihood tree was constructed from the orthologous gene sets (Fig. 3), revealing that *P. perniciosus* clusters most closely with *P. argentipes*. This clustering could be influenced by their similar genome sizes (~97.6 Mb). Given that *P. argentipes* is the primary vector of *L. donovani* and *P. perniciosus* is a major vector of *L. infantum*, this close genomic relationship may reflect shared molecular determinants of vector competence for visceral leishmaniasis parasites, despite their differing geographic distributions. Previously, various molecular markers (ITS, COX1) also showed that *P. (Larrossius) perniciosus* is closer to *P. (Euphlebotomus) argentipes* than to *P. (Phlebotomus) papatasi*. The high similarity between *P. perniciosus* and other *Phlebotomus* species, including *P. argentipes* and *P. papatasi*, further confirms the robustness of the assemblies and reflects their close evolutionary relationships within the Phlebotominae subfamily.

**Table 3:** Ortholog analysis.

	*Lu. longipalpis*	*P. argentipes*	*P. papatasi*	*P. perniciosus Canu*	*P. perniciosus Flye*	*S. squamirostris*
No. genes	20287	15698	20318	17962	17887	15026
Genes in orthogroups (%)	96.9	98.1	97.4	97.7	97.8	89.8
Unassigned genes (%)	3.1	1.9	2.6	2.3	2.2	10.2
Orthogroups containing species (%)	88.1	85.1	88.3	86.0	86.2	85.8
Genes in species-specific orthogroups (%)	4.2	1.1	3.5	3.4	2.9	9.3

Comparing the proteins of the two assemblies against known existing proteins of closely related species. Analysis performed using OrthoFinder software.

Maximum-likelihood phylogenetic trees generated from gene orthologs across sand fly genomes for (A) the Canu assembly and (B) the Flye assembly, including *S. squamirostris*, *Lu. longipalpis*, *P. papatasi*, *P. argentipes*, and *P. perniciosus*. (C) Bar plot showing species-specific and non-species specific orthogroups.

### Mitochondrial analysis

**Fig. 4:**
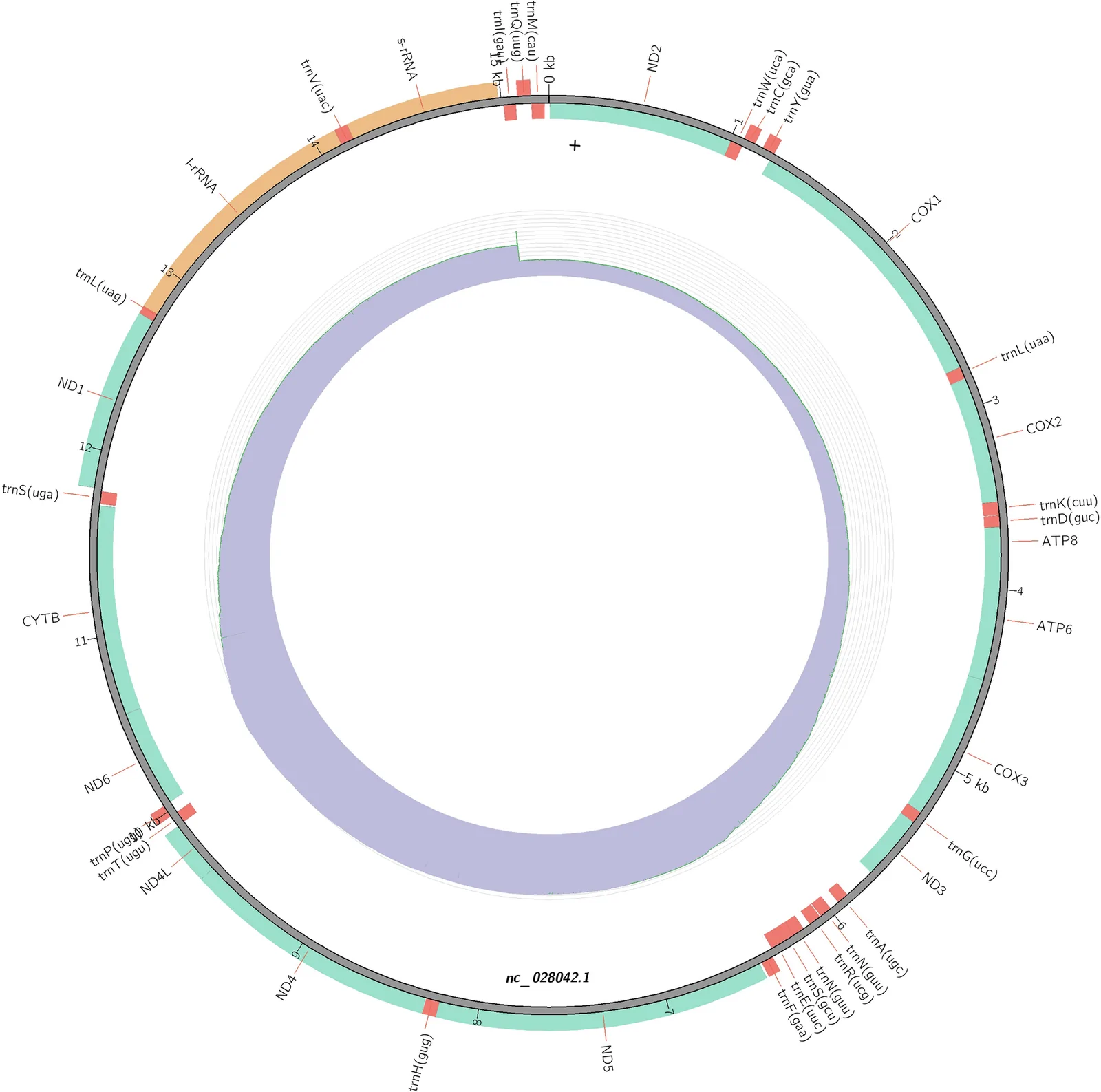
The mitochondrial genome of *P. perniciosus*. A circular mitochondrial genome of *P. perniciosus*, annotated using the MitoZ software, with coverage shown in the centre.

The mitochondrial genome of *P. perniciosus* is 15,253 bp in length and annotated with tRNA and rRNA genes (23 tRNA and 2rRNA) (Fig. 4, Supplementary Table 2). The order of the genes also matches the guided assembly of *P. papatasi*, indicating a conserved mitochondrial gene arrangement within the genus. The annotated tRNA genes show the characteristic secondary structures, which are essential for mitochondrial translation, and no major deviations from insect mitochondrial tRNAs were observed. The rRNA genes also align well with those of *P. papatasi*, suggesting functional conservation. The circular mitochondrial genome exhibits a GC content that is consistent with related species, with a GC content of 21.84% for *P. perniciosus* and 22.14% for *P. papatasi*.

Although large-scale phylogenetic comparisons with other *Phlebotomus* species are limited by available sequences, the consistency in mitochondrial genome structure provides additional confidence in the assembly quality as it validates the raw data that was used to produce the mitochondrial sequence.

## Supplementary information

Supplementary figures and tables

## Data Availability

Custom scripts were used only for generating figures and plots. All other analyses were performed using standard bioinformatic tools described in the Methods section, with tool-specific parameters reported where relevant.

## References

[1] Dvorak, V., Shaw, J. & Volf, P. Parasite Biology: The Vectors. *in* Bruschi F, Gradoni L (eds.), *The Leishmaniases: Old Neglected Tropical Diseases*. Springer, pp 31–77 (2018).

[2] Alten, B. *et al*. Seasonal Dynamics of Phlebotomine Sand Fly Species Proven Vectors of Mediterranean Leishmaniasis Caused by *Leishmania infantum*. *PLoS Neglected Tropical Diseases* **10**, e0004458 (2016). 10.1371/journal.pntd.0004458 PMC4762948 26900688

[3] De Freitas Milagres, T. & Maia, C. *Phlebotomus perniciosus*. *Trends in Parasitology* **40**, 649–650 (2024). 10.1016/j.pt.2024.04.007 38704297

[4] Semenza, J. C. & Paz, S. Climate change and infectious disease in Europe: Impact, projection and adaptation. *The Lancet Regional Health - Europe* **9**, 100230 (2021). 10.1016/j.lanepe.2021.100230 PMC8513157 34664039

[5] Rodríguez-Escolar, I. *et al*. Analysis of the current risk of *Leishmania infantum* transmission for domestic dogs in Spain and Portugal and its future projection in climate change scenarios. *Frontiers in Veterinary Science* **11** (2024). 10.3389/fvets.2024.1399772 PMC11096601 38756515

[6] Ye, F., Liu, T., King, S. D. & You, P. Mitochondrial genomes of two phlebotomine sand flies, *Phlebotomus chinensis* and *Phlebotomus papatasi* (Diptera: Nematocera), the first representatives from the family Psychodidae. *Parasites & Vectors* **8** (2015). 10.1186/s13071-015-1081-1 PMC4573934 26381614

[7] Giraldo-Calderón, G. I. *et al*. VectorBase.org updates: bioinformatic resources for invertebrate vectors of human pathogens and related organisms. *Current Opinion in Insect Science* **50**, 100860 (2021). 10.1016/j.cois.2021.11.008 PMC9133010 34864248

[8] Huang, M. *et al*. Improved high quality sand fly assemblies enabled by ultra low input long read sequencing. *Scientific Data* **11** (2024). 10.1038/s41597-024-03628-y PMC11344823 39181902

[9] Itokawa, K. *et al*. Genomic analysis for co-infecting *Wolbachia* and *Candidatus Tisiphia* the sand fly, *Sergentomyia squamirostris*. *Frontiers in Microbiology* **16** (2025). 10.3389/fmicb.2025.1577636 PMC12123380 40454368

[10] Labbé, F. *et al*. Genomic analysis of two phlebotomine sand fly vectors of Leishmania from the New and Old World. *PLoS Neglected Tropical Diseases* **17**, e0010862 (2023). 10.1371/journal.pntd.0010862 PMC10138862 37043542

[11] Martin-Martin, I., Aryan, A., Meneses, C., Adelman, Z. N. & Calvo, E. Optimization of sand fly embryo microinjection for gene editing by CRISPR/Cas9. *PLoS Neglected Tropical Diseases* **12**, e0006769 (2018). 10.1371/journal.pntd.0006769 PMC6150542 30180160

[12] Kykalová, B. V., Sassù, F., Volf, P. & Telleria, E. L. RNAi-mediated gene silencing of *Phlebotomus papatas*i defensins favors Leishmania major infection. *Frontiers in Physiology* **14** (2023). 10.3389/fphys.2023.1182141 PMC10230645 37265840

[13] Volf, P. & Volfova, V. Establishment and maintenance of sand fly colonies. *Journal of Vector Ecology* **36**, S1–S9 (2011). 10.1111/j.1948-7134.2011.00106.x 21366760

[14] *NCBI Sequence Read Archive* https://www.ncbi.nlm.nih.gov/sra?term=SRP702036 (2026).

[15] Lawyer, P., Killick-Kendrick, M., Rowland, T., Rowton, E. & Volf, P. Laboratory colonization and mass rearing of phlebotomine sand flies (Diptera, Psychodidae). *Parasite* **24**, 42 (2017). 10.1051/parasite/2017041 PMC5687099 29139377

[16] Wood, D. E., Lu, J. & Langmead, B. Improved metagenomic analysis with Kraken 2. *Genome Biology* **20** (2019). 10.1186/s13059-019-1891-0 PMC6883579 31779668

[17] Marijon, P., Chikhi, R. & Varré, J.-S. yacrd and fpa: upstream tools for long-read genome assembly. *Bioinformatics* **36**, 3894–3896 (2020). 10.1093/bioinformatics/btaa262 32315402

[18] De Coster, W. & Rademakers, R. NanoPack2: population-scale evaluation of long-read sequencing data. *Bioinformatics* **39** (2023). 10.1093/bioinformatics/btad311 PMC10196664 37171891

[19] Koren, S. *et al*. Canu: scalable and accurate long-read assembly via adaptivek-mer weighting and repeat separation. *Genome Research* **27**, 722–736 (2017). 10.1101/gr.215087.116 PMC5411767 28298431

[20] Kolmogorov, M., Yuan, J., Lin, Y. & Pevzner, P. A. Assembly of long, error-prone reads using repeat graphs. *Nature Biotechnology* **37**, 540–546 (2019). 10.1038/s41587-019-0072-8 30936562

[21] Coombe, L., Warren, R. L., Wong, J., Nikolic, V. & Birol, I. NTLink: A toolkit for de novo genome assembly scaffolding and mapping using long reads. *Current Protocols* **3** (2023). 10.1002/cpz1.733 PMC10091225 37039735

[22] Alonge, M. *et al*. Automated assembly scaffolding using RagTag elevates a new tomato system for high-throughput genome editing. *Genome Biology* **23** (2022). 10.1186/s13059-022-02823-7 PMC9753292 36522651

[23] Kreutzer, R. D., Modi, G. B., Tesh, R. B. & Young, D. G. Brain cell karyotypes of six species of new and old world sand flies (Diptera: psychodidae). *Journal of Medical Entomology* **24**, 609–612 (1987). 10.1093/jmedent/24.6.609 3694625

[24] Troiano, G. The karyotype of *Phlebotomus perniciosus* with some observations on cytogenetics of Phlebotominae in relation to the other Psychodidae. *Parassitologia* **24**, 231–236 (1982). 6926940

[25] Xu, M. *et al*. TGS-GapCloser: A fast and accurate gap closer for large genomes with low coverage of error-prone long reads. *GigaScience* **9** (2020). 10.1093/gigascience/giaa094 PMC7476103 32893860

[26] Vaser, R., Sović, I., Nagarajan, N. & Šikić, M. Fast and accurate de novo genome assembly from long uncorrected reads. *Genome Research* **27**, 737–746 (2017). 10.1101/gr.214270.116 PMC5411768 28100585

[27] Marçais, G. & Kingsford, C. A fast, lock-free approach for efficient parallel counting of occurrences of k-mers. *Bioinformatics* **27**, 764–770 (2011). 10.1093/bioinformatics/btr011 PMC3051319 21217122

[28] Ranallo-Benavidez, T. R., Jaron, K. S. & Schatz, M. C. GenomeScope 2.0 and Smudgeplot for reference-free profiling of polyploid genomes. *Nature Communications* **11** (2020). 10.1038/s41467-020-14998-3 PMC7080791 32188846

[29] Simão, F. A., Waterhouse, R. M., Ioannidis, P., Kriventseva, E. V. & Zdobnov, E. M. BUSCO: assessing genome assembly and annotation completeness with single-copy orthologs. *Bioinformatics* **31**, 3210–3212 (2015). 10.1093/bioinformatics/btv351 26059717

[30] Guan, D. *et al*. Identifying and removing haplotypic duplication in primary genome assemblies. *Bioinformatics* **36**, 2896–2898 (2020). 10.1093/bioinformatics/btaa025 PMC7203741 31971576

[31] Wick, R. R. & Holt, K. E. Polypolish: Short-read polishing of long-read bacterial genome assemblies. *PLoS Computational Biology* **18**, e1009802 (2022). 10.1371/journal.pcbi.1009802 PMC8812927 35073327

[32] Li, H. & Durbin, R. Fast and accurate short read alignment with Burrows–Wheeler transform. *Bioinformatics* **25**, 1754–1760 (2009). 10.1093/bioinformatics/btp324 PMC2705234 19451168

[33] Li, H. *et al*. The Sequence Alignment/Map format and SAMtools. *Bioinformatics* **25**, 2078–2079 (2009). 10.1093/bioinformatics/btp352 PMC2723002 19505943

[34] Cingolani, P. *et al*. A program for annotating and predicting the effects of single nucleotide polymorphisms, SnpEff. *Fly* **6**, 80–92 (2012). 10.4161/fly.19695 PMC3679285 22728672

[35] Tarailo‐Graovac, M. & Chen, N. Using RepeatMasker to identify repetitive elements in genomic sequences. *Current Protocols in Bioinformatics* **25** (2009). 10.1002/0471250953.bi0410s25 19274634

[36] Flynn, J. M. *et al*. RepeatModeler2 for automated genomic discovery of transposable element families. *Proceedings of the National Academy of Sciences* **117**, 9451–9457 (2020). 10.1073/pnas.1921046117 PMC7196820 32300014

[37] Shumate, A. & Salzberg, S. L. Liftoff: accurate mapping of gene annotations. *Bioinformatics* **37**, 1639–1643 (2020). 10.1093/bioinformatics/btaa1016 PMC8289374 33320174

[38] Brůna, T. *et al*. Galba: genome annotation with miniprot and AUGUSTUS. *BMC Bioinformatics* **24** (2023). 10.1186/s12859-023-05449-z PMC10472564 37653395

[39] Gabriel, L. *et al*. BRAKER3: Fully automated genome annotation using RNA-seq and protein evidence with GeneMark-ETP, AUGUSTUS and TSEBRA. *bioRxiv (Cold Spring Harbor Laboratory)*, 10.1101/2023.06.10.544449 (2023). PMC11216308 38866550

[40] Chan, P. P., Lin, B. Y., Mak, A. J. & Lowe, T. M. tRNAscan-SE 2.0: improved detection and functional classification of transfer RNA genes. *Nucleic Acids Research* **49**, 9077–9096 (2021). 10.1093/nar/gkab688 PMC8450103 34417604

[41] Seemann, T. barrnap 0.9: rapid ribosomal RNA prediction. *GitHub repository* https://github.com/tseemann/barrnap (2014).

[42] Fu, L., Niu, B., Zhu, Z., Wu, S. & Li, W. CD-HIT: accelerated for clustering the next-generation sequencing data. *Bioinformatics* **28**, 3150–3152 (2012). 10.1093/bioinformatics/bts565 PMC3516142 23060610

[43] Tang, H. *et al*. JCVI: A versatile toolkit for comparative genomics analysis. *iMeta* **3** (2024). 10.1002/imt2.211 PMC11316928 39135687

[44] Wang, Y. *et al*. MCScanX: a toolkit for detection and evolutionary analysis of gene synteny and collinearity. *Nucleic Acids Research* **40**, e49 (2012). 10.1093/nar/gkr1293 PMC3326336 22217600

[45] Kurtz, S. *et al*. Versatile and open software for comparing large genomes. *Genome Biology* **5** (2004). 10.1186/gb-2004-5-2-r12 PMC395750 14759262

[46] Ponnusamy, H. *pyCircos*: a Python library for Circos plots. *GitHub repository* https://github.com/ponnhide/pyCircos (2023).

[47] Emms, D. M. & Kelly, S. OrthoFinder: phylogenetic orthology inference for comparative genomics. *Genome Biology* **20** (2019). 10.1186/s13059-019-1832-y PMC6857279 31727128

[48] Katoh, K. & Standley, D. M. MAFFT Multiple Sequence Alignment Software Version 7: Improvements in performance and usability. *Molecular Biology and Evolution* **30**, 772–780 (2013). 10.1093/molbev/mst010 PMC3603318 23329690

[49] Price, M. N., Dehal, P. S. & Arkin, A. P. FastTree 2 – approximately Maximum-Likelihood trees for large alignments. *PLoS ONE* **5**, e9490 (2010). 10.1371/journal.pone.0009490 PMC2835736 20224823

[50] Letunic, I. & Bork, P. Interactive Tree Of Life (iTOL) v5: an online tool for phylogenetic tree display and annotation. *Nucleic Acids Research* **49**, W293–W296 (2021). 10.1093/nar/gkab301 PMC8265157 33885785

[51] Shen, W., Le, S., Li, Y. & Hu, F. SeqKit: a Cross-Platform and ultrafast toolkit for FASTA/Q file manipulation. *PLoS ONE* **11**, e0163962 (2016). 10.1371/journal.pone.0163962 PMC5051824 27706213

[52] Li, H. *seqtk*: a fast and lightweight tool for processing sequences in FASTA/Q formats. GitHub repository https://github.com/lh3/seqtk (2012).

[53] Camacho, C. *et al*. BLAST+: architecture and applications. *BMC Bioinformatics* **10** (2009). 10.1186/1471-2105-10-421 PMC2803857 20003500

[54] Meng, G., Li, Y., Yang, C. & Liu, S. MitoZ: a toolkit for animal mitochondrial genome assembly, annotation and visualization. *Nucleic Acids Research* **47**, e63 (2019). 10.1093/nar/gkz173 PMC6582343 30864657

[55] Thorpe, J. *Genbank* https://www.ncbi.nlm.nih.gov/datasets/genome/GCA_056882495.1 (2026).

[56] Thorpe, J. *Phlebotomus perniciosus* annotation dataset. *Zenodo*10.5281/zenodo.20392245 (2026).

