# Supplementary material for: High quality Chromosome-scale Genome Assembly of Phlebotomus perniciosus, a Vector of Zoonotic Leishmaniasis
Source: Sci Data. 2026 Jul 1;13:1471. doi: 10.1038/s41597-026-07617-1 (PMC13687099; doi:10.1038/s41597-026-07617-1)
Supplement: Supplementary file 1 [file 41597_2026_7617_MOESM1_ESM.docx]

**Supplementary materials**

**Supplementary figure 1. The estimated genomes size.** The estimated genome size as calculated using Jellyfish software and GenomeScope2.0 to plot.

**Supplementary figure 2. Snail plot of BUSCO scores.** Snail plot created using the blobtoolkit software. **A.** Canu BUSCO results **B.** Flye BUSCO results.

**Supplementary Table 1. Assembly Performance and Computational Resource Comparison between Canu and Flye.**

|  | Canu | Flye |
| --- | --- | --- |
| Time taken | 844 minutes | 126 minutes |
| N50 | 47Mb | 48MB |
| Busco score | 94.5% | 94.7% |
| Total alignment length | 142461568 | 142614609 |
| Number of scaffolds | 76 | 222 |
| Largest scaffold | 51.9Mb | 51.6MB |
| Percentage gaps | 0.025% | 0.017% |
| N’s per 100kbp | 25.34 | 16.86 |
| Maximum memory used | 64MB | 64MB |
| CPU threads used | 32 | 32 |
| Disk space used | 145G | 4.7G |
| Assembler version used | 2.2 | 2.9.5 |

**Supplementary Table 2. Mitochondria assembly assessment.**

|  | P. perniciosus mitochondria |
| --- | --- |
| Length | 15,253 |
| Circular | Yes |
| Closely related species | *P. papatasi* |
| Assembly software used | Flye |
| Total genes found | 38 |
| Protein coding genes found | 13 |
| tRNA genes found | 23 |
| rRNA genes found | 2 |
| Duplicate genes found | 6 |
